# Meta-Genomic Analysis of Different Bacteria and Their Genomes Found in Raw Buffalo Milk Obtained in Various Farms Using Different Milking Methods

**DOI:** 10.3390/genes15081081

**Published:** 2024-08-15

**Authors:** Ling Li, Wenhao Miao, Zhipeng Li, Li Huang, Enghuan Hau, Muhammad Farhan Khan, Qingyou Liu, Qingkun Zeng, Kuiqing Cui

**Affiliations:** 1 State Key Laboratory for Conservation and Utilization of Subtropical Agro-Bioresources, Guangxi University, Nanning 530004, China; lling2010@163.com (L.L.); percenatania@gmail.com (W.M.); zp.li@gxu.edu.cn (Z.L.); qyliu-gene@fosu.edu.cn (Q.L.); 2 Guangxi Zhuang Autonomous Region Buffalo Milk Quality and Safety Control Technology Engineering Research Center, Guangxi Buffalo Research Institute, Chinese Academy of Agricultural Sciences, Nanning 530001, China; huangli00206@163.com (L.H.); enghuan_90@yahoo.com (E.H.); 3 Department of Chemistry, Gomal University, Dera Ismail Khan 29050, Pakistan; farhankhanbgu@gmail.com; 4 Guangdong Provincial Key Laboratory of Animal Molecular Design and Precise Breeding, School of Life Science and Engineering, Foshan University, Foshan 528225, China

**Keywords:** raw buffalo milk, metagenomics, psychrophile

## Abstract

Milking methods have significant impacts on the microbiological composition, which could affect the quality of raw buffalo milk. Hence, the current study was conducted on the impact of milking methods on microorganisms in buffalo tank raw milk from 15 farms in Guangxi, China. The farms were divided into two groups based on the milking method: mechanical milking (MM, *n* = 6) and hand milking (HM, *n* = 9). Somatic cell counts, bacterial cell counts and nutrients of the raw buffalo milk samples were analyzed. The comparison of raw buffalo milk samples was analyzed using metagenomic sequencing to detect any differences between the two groups. There was no significant difference in the basic nutritional compositions and somatic cell count of raw buffalo milk between the two milking methods. However, the HM samples had significantly higher bacterial counts and diversity compared to the MM samples. The results showed that *Staphylococcus* spp., *Klebsiella* spp., *Streptococcus* spp., and *Pseudomonas* spp. were the major microbes present in canned raw buffalo milk. However, the differences between the two milking methods were the relative abundance of core microorganisms and their potential mastitis-causing genera, including the content of antibiotic-resistance genes and virulence genes. Our study revealed that *Staphylococcus* spp. and *Streptococcus* spp. were significantly more abundant in the MM group, while *Klebsiella* spp. was more abundant in the HM group. Regardless of the milking method used, *Pseudomonas* spp. was identified as the primary genus contributing to antibiotic resistance and virulence genes in canned raw buffalo milk. These findings affirm that there are differences in the microbial and genomic levels in canned raw milk. To prove the functional roles of the discovered genes and how these genes affect milk quality, further research and experimental validation are necessary.

## 1. Introduction

Buffalo milk, with its intriguing nutritional profile, is attracting investments and research attention worldwide [1]. In southern China, Southeast Asia, and some European countries, buffalo milk and its products have a positive impact on the local population as a source of protein, vitamins, and minerals [2]. However, the high nutrient content in raw buffalo milk also creates an ideal environment for bacterial growth and development. Microorganisms have not only an important impact on the pricing and quality of dairy products but also on the financial performance of business corporates [3]. Microbial colonization in milk poses a major threat to both product quality and human health [4]. Currently, dairy buffalo farming in Guangxi exists in two modes: large-scale farms and small-scale free-range farming. Raw buffalo milk is purchased centrally by milk stations, and then transported to processing enterprises in a cold chain. Milking methods can have significant impacts on the microbiological compositions and qualities of raw buffalo milk.

In addition to its native microbiota, milk promotes the growth of microorganisms from animal surfaces and the surrounding environment [5]. Three primary sources of microbial contamination of bulk milk at the farm level are bacteria from the outer layer of the udder and teats, microorganisms from the surface of the milking apparatus, and organisms that cause mastitis from inside the udder [6]. Furthermore, mastitis in cows can be caused by environmental pathogens that enter through the teat, where the common bacteria are *Escherichia coli*, *Klebsiella* spp., *Streptococcus lactis*, and *Streptococcus uberis* [7], at which the presence of these bacteria in high yielding cows could result in significant economic losses [8]. Mastitis can be classified into two forms: clinical and subclinical. Previous literature reports that the global prevalence of clinical and subclinical mastitis in dairy buffaloes was 28% and 46%, respectively [9]. Moreover, mastitis reduces milk production and increases the risk of culling in buffalos [10].

After milking, raw buffalo milk is stored at a low temperature of 4 °C and transported to the processing plant via a cold chain. It has been demonstrated in the literature that low temperatures inhibit the growth of most bacteria, but cryophilic bacteria in milk continue to grow and produce heat-stable proteases, which are capable of hydrolyzing casein micelles, subsequently affecting the quality of the dairy product [11]. According to a previous study, *Pseudomonas* spp. and *Acinetobacter* spp. dominated raw buffalo milk with a combined relative abundance of over 60%, after 72 h of storage [12]. Therefore, monitoring *Pseudomonas* spp. in raw milk has become an essential aspect of product quality control.

Despite continuous updates on farming techniques and treatments, the treatment of mastitis in dairy cows depends heavily on antibiotic administration [10]. In May 2023, the World Organization for Animal Health (WOAH) revealed that global quantities of antimicrobials intended for use in animals were 114.5 million tonnes, 91.11 million tonnes, and 96.73 million tonnes in 2017, 2018, and 2019, respectively. In the past three years, the use of antibiotics in animals has increased by 45% in African countries and 5% in the whole of America [13]. However, the continuous use of antibiotics leads to mutations in the bacteria and gradual buildup of multidrug resistance genes in their genomes, which lead to the development of resistant phenotypes that have the capacity to survive under the higher pressure of antibiotics. Moreover, it is probable that the survival of bacteria resistant to different drugs may be significantly influenced by variations in the use of various clinical and nonclinical environments. Following the emergence of co-selection, this persistence causes co-resistance to proliferate in the surrounding environment [14]. Similarly, many genes associated with virulence factor genes (VFGs) can be transferred horizontally through mobile genetic elements such as plasmids, gene islands, and phages. The role of various VFGs targeted in bacterial mastitis pathogens has been extensively studied [15,16,17,18]. However, there is still a lack of research conducted on the buffalo milk microbiome. Raw buffalo milk, as an animal product, serves as the intersection for the horizontal and vertical transmission of antibiotic resistance genes and virulence genes in the environment and individual buffaloes. The enrichment of these genes in raw buffalo milk may pose a new risk. A systematic understanding of the structure of antibiotic resistance genes and virulence genes in raw buffalo milk, as well as their relationship with milking methods, can enhance our knowledge and education. This can increase awareness of antibiotic resistance and bacterial virulence, and also provide a guide on production practices.

The amount of microbe and its compositions in raw buffalo milk can affect the quality of the final dairy product and, subsequently, affect human and animal health. This study aims to evaluate the types and distribution of microorganisms, as well as their genome compositions, in raw buffalo milk harvested using different milking methods. High-throughput sequencing was used to analyze the number and distribution characteristics of microorganisms within the milk. This study aims to ensure better quality of dairy products by understanding the occurrence and spread of pathogens in raw buffalo milk. Furthermore, it can increase employees’ awareness of safety measures and decrease health hazards in work settings, such as animal housing.

## 2. Materials and Methods

### 2.1. Sample Collection

Fifteen raw buffalo milk samples were collected in 2022 from five major buffalo milk-producing areas in Guangxi (China), including Nanning, Lingshan, and Hengxian. There were six from mechanically milked (MM) large-scale farms and nine from hand-milked (HM) farms. The samples were collected from each tank and divided into sample sizes after collection. The samples for macro-genomic sequencing were immediately stored on dry ice and transported to Shanghai Biotechnology Co., Ltd. (Shanghai, China). Other milk samples were kept at low temperature and transported back to the laboratory for further analyses including milk compositions, somatic cell count (SCC) and total number of bacterial cells (analyzed using a BacSomatic™ instrument (FOSS Analytical, Hillerød, Denmark)). The compositions of milk including milk protein, fat, total milk solids, nonfat milk solids and lactose content, were analyzed using a MilkScan™ FT 3 Milk Composition Meter (FOSS Analytical, Hillerød, Denmark).

### 2.2. DNA Extraction

The milk sample is a low-biomass material. The potential contaminants in low-biomass samples during the experimental process of sequencing-based studies could produce biased results. To address these concerns, we implemented several measures to minimize contamination and ensure the accuracy of our results. All equipment and reagents were sterilized prior to use, and samples were handled in a laminar flow hood to reduce the risk of airborne contaminants. DNA from raw buffalo milk samples was extracted as previously described [19]. A 15 mL of the milk sample was centrifuged at 4500× *g* for 20 min at 4 °C, followed by 13,000× *g* for 1 min at 4 °C for 2 times (Eppendorf, Hamburg, Germany). After each centrifugation step, the supernatant was aseptically removed, and sterile phosphate-buffered saline (PBS) was used to wash the retained pellet. The pellet was used for DNA extraction using the E.Z.N.A. Soil DNA Kit (Omega Bio-Tek, Norcross, GA, USA), following the manufacturer’s guidelines. We measured the DNA concentration using a Qubit 4.0 (Thermo Fisher Scientific, Waltham, MA, USA) and constructed the sample library using VAHTS Universal Plus DNA Library Pren Kit for Illumina. The library underwent fragment quality inspection with Qsep-400, and its concentration was measured using Qubit 3.0. Finally, the constructed library was sequenced with the Illumina NovaSeq 6000 (Illumina, San Diego, CA, USA).

### 2.3. Metagenomic Sequencing

The library’s fragment quality was assessed using the Qsep-400, and its concentration was determined via the Qubit 3.0. Sequencing of the constructed library was executed on the Illumina NovaSeq 6000 platform (Illumina, San Diego, CA, USA). MEGAHIT (Version 1.2.9) software is used for collecting clean reads [20]. Post-inspection of the library fragments by Qsep-400 and concentration measurement with Qubit 3.0, the sequencing process was completed using the Illumina NovaSeq 6000 (Illumina, San Diego, CA, USA) [21]. The non-redundant gene sets were compiled using MMseqs2 [22], applying a sequence similarity threshold of 95% and an alignment coverage threshold of 90% [23].

### 2.4. Taxonomic Assignment, Functional Annotation and Gene Function Analysis

The Nr database is a comprehensive non-redundant protein database that contains detailed information about the corresponding species as well as substantial protein sequences and annotations. Genes were aligned using Blastp to the Nr database to identify the most similar sequences. Based on the matched Nr sequences, the species composition and relative abundance in the raw buffalo milk samples were determined. Public databases such as CARD [24] and VFDB [25] were employed to predict gene functions, using e-values less than 10^−5^ and scores exceeding 60.

### 2.5. Statistical Analysis

α diversity was calculated using QIIME 1.1, and β diversity was assessed using UniFrac distances. α diversity results were expressed as “mean ± standard deviation”, with *p* < 0.05 indicating significant differences. Statistical analyses were conducted using Microsoft Excel 2019 and IBM SPSS Statistics 25 software, and community structure analysis histograms in bacterial diversity were generated in GraphPad Prism 9.5.0. β diversity, which assessed microbiome differences between samples, was visualized using dimensionality reduction methods such as principal coordinate analysis (PCoA), non-metric multidimensional scaling (NMDS), or constrained principal component analysis (PCA). These analyses were visualized using the R vegan software package (version 2.5-6), and the distances between samples were displayed as scatter plots. Other figures were created using the R package ggplot2 v3.3.5.

## 3. Results and Discussion

### 3.1. Effect of Different Milking Methods on the Basal Nutrient Content, SCC and Number of Bacterial Cells of Raw Buffalo Milk

The results showed that there was no significant difference in milk composition and SCC between the two milking methods, although a few farms had a higher SCC in HM. In contrast, there was a significant increase in total number of bacterial cells in HM compared to MM (Table 1). The results were aligned with previous literature, where the MM appears to be highly effective in terms of milking and milk hygiene [26]. Literature suggests that the hygiene of the milker’s hands, parlor, and barn during the milking process influences the number and composition of microorganisms in the final raw milk [27]. Mechanical milking reduced the residence time of teat bacteria in raw buffalo milk through high milking frequency, which reduced the exposure time of raw buffalo milk to air. In conclusion, the differences in the effects of different milking methods on raw milk quality and SCC were not significant, but the effects on total bacterial cells in final buffalo raw milk were more significant.

### 3.2. Milking Methods Influence the Raw Milk Microbiota

**Figure 1:**
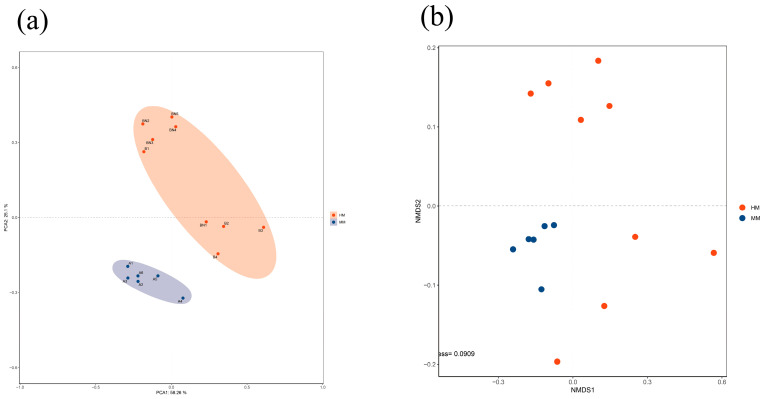
Principal component analysis (PCA) and principal coordinate analysis (PCA) of bacterial communities in raw milk. (**a**) Microbial communities in the HM and MM milk sample pools from β-diversity (principal component analysis; PCA), measured in the Bray–Curtis distance method (genus level). (**b**) Non-metric multidimensional scaling (NMDS) ordination plot with fitted macro-genomic variables (genus level).

An average of 62,336,920 high-quality paired-end reads were generated per sample. An average of 94.7% of metagenomic reads were host genome reads (range 81.9% to 98.7%). This resulted in an average of 3,403,722 reads that were assigned as microbial reads, which can be used for taxonomic and functional characterization (Supplementary Table S1). α diversity refers to the diversity within a specific environment or ecosystem, where it is mainly used to reflect the richness and evenness of species. The diversity is often reflected by Chao1, Observed species, Good’s coverage, Shannon, Simpson, and other indices (Table 2). The results showed that the observed species, Chao1, ACE, Shannon, and Good’s coverage indices reflected that the species richness in the milk of the HM group was significantly higher than that in the MM group. The Good’s coverage results show that uni-genes were abundant in all the samples. As shown in Figure 1a. PCoA plots based on the Bray–Curtis distance method at the genus level indicated that the samples in the MM group were more tightly clustered (yellow circles), while the samples in the HM group were more dispersed (red circles). The NMDS grading plots demonstrated a clear separation of HM and MM milk samples by the Bray–Curtis distance method on the read data at the genus level (Figure 1b). The microbiota associated with MM was tightly clustered on the left side of NMDS1, while the HM milk microbiota was dispersed towards the other side of NMDS1. In addition, the microbiome profiles at the upper genus level between HM and MM milk showed a clear separation (*p* = 0.023), when the two images were combined. There were significant differences in α diversity (chao1, ACE, and Shannon’s index) between HM and MM samples, indicating that the microbial ecosystems of MM milk were more diverse. Previous literature confirmed that raw milk with mastitis (either clinical or subclinical) exhibited a significant increase in species diversity (α diversity) compared to the healthy group, using 16S rRNA-based gene sequencing [28].

### 3.3. Structure and Composition of the Milk Microbiome of Buffaloes with Different Milking Methods

**Figure 2:**
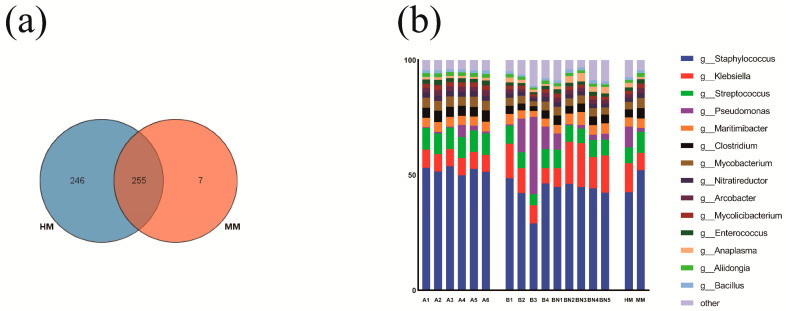
Taxonomic composition of the raw buffalo milk microbiome. (**a**) Venn diagram showing unique and shared bacteria at the genus level (**b**) Taxonomic signatures of relative abundance at the microbial genus level greater than 1% for samples based on macro-genomics.

At the domain level, bacteria were the most abundant community in raw buffalo milk, accounting for approximately 99.85% of the total microorganisms. Fungi accounted for approximately 0.13%, viruses about 0.02%, and archaea were 0.00004% of the total microorganisms, respectively. Though the relative abundance of microbes was higher in HM milk, compared to MM milk, the abundance fluctuated more (CV = 49.9758 vs. 71.3008). Figure 2a shows Venn diagrams of microbial taxa found in HM and MM samples. The study detected 501 bacteria genera in HM and 262 bacterial genera in MM samples. A total of 255 genera (50.20%) were detected in both genomes (Figure 2a). The results showed that there was a significant difference in the microbial composition in raw buffalo milk from HM and MM. Moreover, the relative abundance analysis of the macro-genomes showed that the four most abundant phyla were *Firmicutes*, *Proteobacteria*, *Actinobacteria* and *Bacteroidetes* (about 99.4% of the total sequences). Furthermore, the genera with an average relative abundance greater than 1% in the final results were *Staphylococcus* spp., *Klebsiella* spp., *Streptococcus* spp., *Pseudomonas* spp., *Maritimibacter* spp., *Clostridium* spp., *Mycobacterium* spp., *Nitratireductor* spp., *Arcobacter* spp., *Mycolicibacterium* spp., *Enterococcus* spp., *Anaplasma* spp., *Aliidongia* spp. and *Bacillus* spp. (Figure 2b). The result showed that metagenomic increased the discriminatory power of this cutting-edge technology in identifying taxa in the milk microbiome [29,30], where the core bacteria *Staphylococcus* spp., *Pseudomonas* spp. and *Bacillus* spp. of raw buffalo milk have been discussed elsewhere in the literature, and the highest abundance of *Staphylococcus* spp. and *Streptococcus* spp. as the main bacterial genera in the milk trucks were similar to the results from the existing assay [31,32].

**Figure 3:**
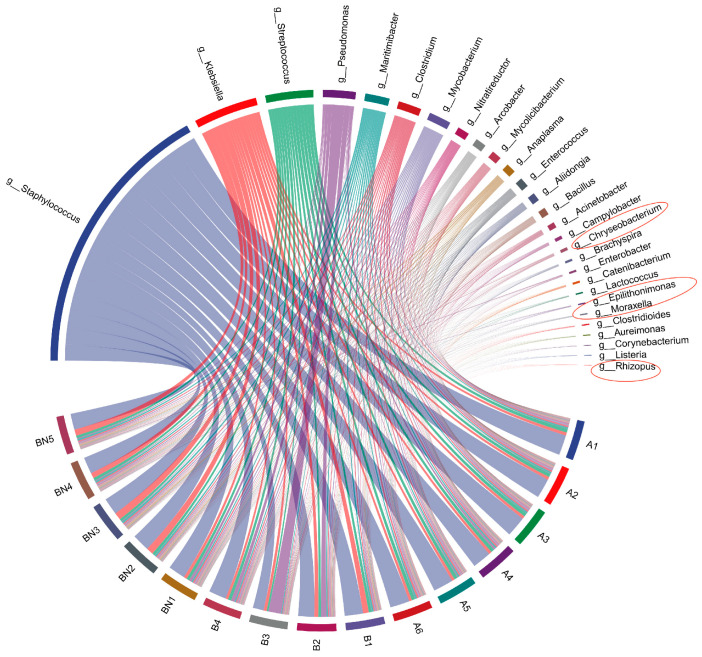
Composition of core microbiota of raw buffalo milk and chord diagrams (Red circles indicate unique micro-organisms at genus level with different milking methods).

Reconstruction of the “core” milk microbiota, i.e., the bacterial taxonomic units shared by specific groups of samples, allows the identification of the most common bacterial species in cow milk. In this case, the “core” microbiota of milk samples was compared between different milking methods, in order to identify the effect of these methods on the most common bacterial species in milk. As mentioned above, only those genera that were more than 75% in the samples, with an average relative abundance of at least more than 0.1% were considered as a part of the “core” milk community [33]. The results showed that a total of 27 genera were found in HM and 25 genera in MM (Figure 3). *Rhizobium* spp. being the specific genus in MM. The genera *Moraxella* spp., *Epilithonimonas* spp. and *Chryseobacterium* spp. were the core bacterial genera specific to HM. *Rhizopus* spp. causes food contamination during storage, transport and storage, and the high temperatures and humidity required for *Rhizopus* spp. to grow are more difficult to achieve, but it is important to be aware of fungal contamination during subsequent handling [34]. *Moraxella* spp. is a genus of aerobic or partially anaerobic fermentative Gram-negative bacilli in the family *Moraxellaceae* of the order *Pseudomonadales*, and there is literature on *Moraxella* spp., *Epilithonimonas* spp. and *Chryseobacterium* spp. detected as part of the psychrophile from raw milk [35]. Therefore, the growth of the relevant psychrophilic bacteria should be closely monitored during refrigerated transport to avoid them becoming dominant species due to the low-temperature environment. The remaining 24 genera were prevalent in both sets of samples, suggesting that these microbial species are typical colonizers of buffalo’s milk, where to some extent, the presence of these species was not related to the milking method. To gain a deeper understanding of the differences in key microbial genera between milking methods, a differential analysis of relative abundance was conducted to determine the variability of bacterial genera between milking practices.

### 3.4. Differences in the Relative Abundance Composition of Core Microbiota and Bacteria Potentially Associated with Mastitis at the Genus Level between Milking Methods

**Figure 4:**
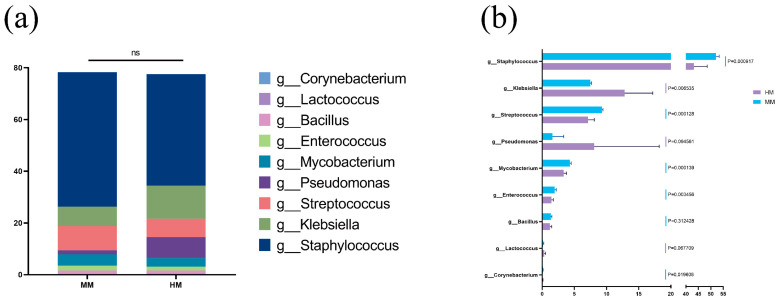
Differences in the relative abundance composition of core microbiota and bacteria potentially associated with mastitis at the genus level between milking methods. (**a**). Stacked histogram of bacterial core genera associated with mastitis in different milking practices; (**b**). Histogram of differences in bacterial core genera associated with mastitis in different milking methods.

The differences in the relative abundance of bacteria at the genus level between HM and MM samples were further investigated to understand the potential impact of the microbiota in raw buffalo milk on mastitis. The conventional causative organisms of bovine mastitis were summarized in Laboratory Handbook on Bovine Mastitis, 3rd Edition, published by the National Mastitis Council (NMC) [36], mainly as *Streptococcus* spp., *Staphylococcus* spp., *Enterococcus* spp., *Lactococcus* spp., *Klebsiella* spp., *Enterobacter* spp., *Salmonella* spp., *Pseudomonas* spp., *Aspergillus* spp., *Pasteurella* spp., *Mycoplasma* spp., *Nocardia* spp., *Prochlorococcus* spp., *Corynebacterium* spp., *Tropezia* spp., *Mycobacterium* spp. and *Bacillus* spp.. Our results showed that there was no significant difference in the total relative abundance of the main bacteria responsible for mastitis in HM and MM samples (Figure 4a). However, the relative abundance of individual bacteria including *Staphylococcus* spp. (*p* = 0.0000917), *Streptococcus* spp. (*p* = 0.000128), *Mycobacterium* spp. (*p* = 0.000139), *Enterococcus* spp. (*p* = 0.003456) and *Corynebacterium* spp. (*p* = 0.019608) were significantly higher in the mechanically milked samples than in the manually milked samples (Figure 4b). This is because the teat canal is opened after milking using high-frequency suction in mechanical milking. Moreover, a higher number of microbes in MM compared to HM could be due to stagnant fluids such as residual milk in the suction which may increase the bacterial counts in raw milk, and contribute to bacterial enrichment of the final tanks [37]. Our findings were aligned with the previous literature which proved that *Staphylococcus* spp. and *Streptococcus* spp. in breastmilk significantly increased with the usage of breast pumps [38].

On the other hand, *Klebsiella* spp. was significantly higher in HM (*p* = 0.006535) than in MM. Moreover, the mean of *Pseudomonas* spp. count was higher in HM than in MM, although the difference was not significant (*p* = 0.094561) (Figure 4b). This result is consistent with the trend of previous studies that *Klebsiella* spp. had a high distribution in HM samples, and *Streptococcus* spp. and *Staphylococcus* spp. were also widely distributed within MM samples [39,40]. The study found that *Klebsiella* spp. were mainly derived from faeces on bedding and associated equipment, particularly organic bedding such as sawdust [41]. The hygiene of the milking parlor as well as the cleaning and feeding facilities of the cows in MM greatly reduces the contact spread of this bacterium. In addition, hygiene conditions have a strong influence on the relative abundance of psychrophiles, which can reach up to 75% in unhygienic conditions [42]. A recent study applied 16S rDNA sequencing to raw milk samples collected over 12 consecutive months and identified Pseudomonas spp. as the most common genus [43]. Although *Pseudomonas* spp. as a psychrophile did not differ in t-test results between milking methods (*p* = 0.094561), the mean value for HM was almost four times higher than that for MM. Previous studies showed that identified bath cups, teat skin and milkers’ hands as the main sources of *Pseudomonas* spp. in raw milk [44]. Therefore, in HM environments, it is important to focus on controlling *Pseudomonas* spp. contamination. The types of flora that require attention vary depending on the milking method used. It is recommended to enhance the control of relevant flora based on the differences in milking methods.

### 3.5. Effect of Milking Methods on Antibiotic Resistance Genes

**Figure 5:**
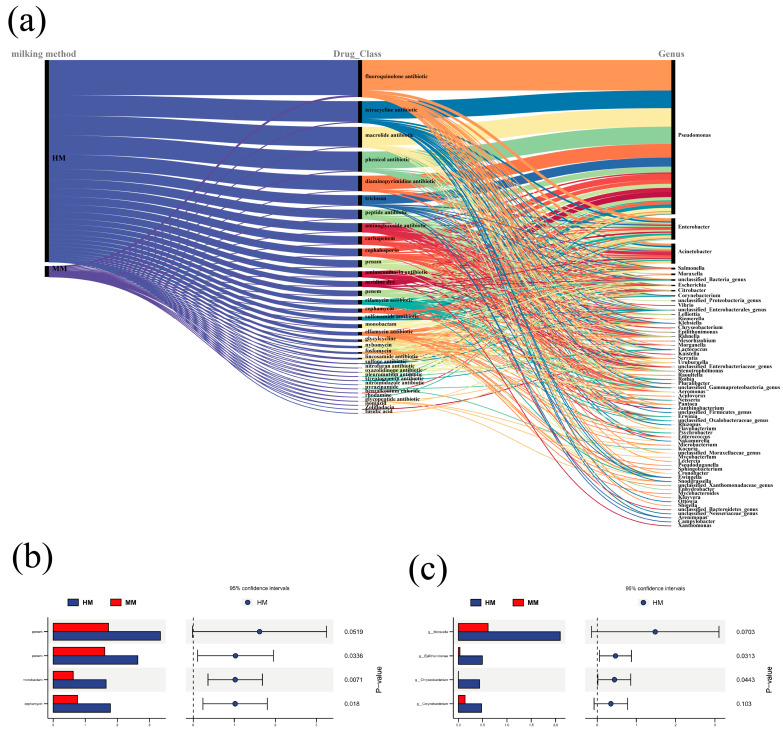
Effect of Milking Methods on Antibiotic resistance genes. (**a**). Alluvial plot of genus level of antibiotic resistance for different milking practices. (**b**). STAMP plot of antibiotic resistance for different milking practices. (**c**). STAMP plot of genus-level classification of antibiotic resistance genes for different.

The relationship between drug resistance of microorganisms and different milking styles is illustrated in Figure 5. The level of bacterial antibiotic resistance genes in HM was much higher than that of MM, but the *t*-test difference was not significant (*p* = 0.0838). The most dominant carriers of antibiotic resistance genes were the genera *Pseudomonas* spp., *Enterobacter* spp., and *Acinetobacter* spp., as determined by corresponding the antibiotic resistance genes to the level of bacterial genera. The main types of antibiotics targeted by antibiotic-resistance genes were Fluoroquinolone, Tetracycline, Macrolide, Phenicol, and Diaminopyrimidine antibiotics. The comparison of antibiotic resistance in raw buffalo’s milk using different milking methods and the reads of their host bacteria were analyzed using statistical analysis of metagenomic profiles (STAMP). The results showed that the four β-lactam antibiotics, including penam, penem, monobactam and cephamycin, had significant differences between the different milking methods (Figure 5b). The genera *Epilithonimonas* spp. and *Chryseobacterium* spp. showed significant differences in antibiotic resistance between different milking methods, hence these genera should be given special attention during the HM process (Figure 5c). This is because the microbiota in animal products is influenced by human bacterial flora during processing or consumption. Antibiotics used for disease control in livestock often share active ingredients with human drugs. Therefore, antibiotic resistance genes accumulated from high levels of antibiotic usage in animal agriculture may be transferred to the human microbiota via animal products. The spread of antibiotic resistance genes may further reduce the efficacy of antibiotic therapy, at which new multi-drug resistant strains may be developed over time [45]. The widespread colonization of raw milk with *Pseudomonas* spp. and *Enterobacter* spp. and their strong correlation with antibiotic resistance genes requires us to pay more attention to the risks and their impact on humans.

### 3.6. Effect of Milking Methods on the Level of Virulence Factors of Pathogenic Bacteria

**Figure 6:**
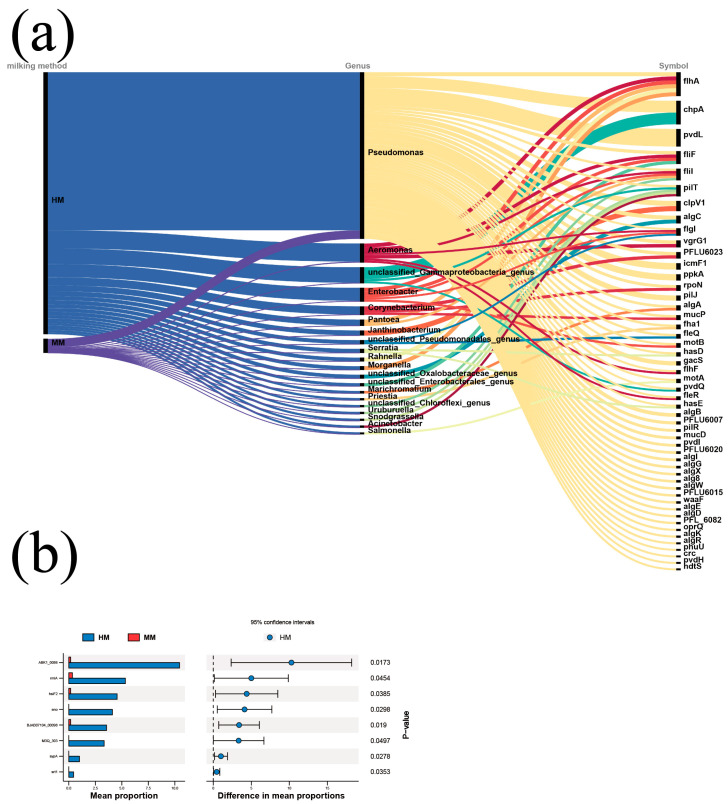
Effect of milking methods on the level of virulence factors of pathogenic bacteria. (**a**). Alluvial plots of virulence factors of pathogenic bacteria at the genus level for different milking styles (top 50 reads). (**b**). STAMP plots of virulence factors of pathogenic bacteria for different milking styles.

The results of the microbial virulence factors of the different milking methods and their correspondence to the bacterial genus level are shown in Figure 6. The amount of virulence genes was much higher in HM than in MM, but the difference was not significant (*p* = 0.1299). The correlation of virulence genes with bacterial genus level showed that *Pseudomonas* spp. and *Aeromonas* spp. were the most dominant carriers of virulence genes. Based on the differential analysis of virulence genes, ABK1_0086, mla, hsiF2, eno, BJAB07104_00098, M3Q_303, sypA and srt1 were the virulence factors that were significantly different between HM and MM. Irrespective of the milking method (Figure 6b), raw buffalo milk is ultimately transported to the processing plant under refrigerated conditions and the virulence factors are associated with the gradual production of extracellular enzymes and exotoxins as *Pseudomonas* spp. and *Aeromonas* spp. multiply. Greater attention should therefore be paid to the effects on milk quality during refrigerated transport.

## 4. Conclusions

Meta-genomic analysis is a complete way to learn about the different kinds of microbes that live in raw buffalo milk. The macro-genomic analysis of raw buffalo milk samples including chemical properties and microbial diversity revealed no significant difference between the samples from different milking methods concerning the major components of milk and somatic cell counts. However, the total number of bacterial cells and microbial diversity were significantly higher in HM than in MM. Moreover, the relative abundance analyses on the core microbiome showed significant differences in microflora between milking methods. Hence, it is important to highlight *Staphylococcus* spp. and *Streptococcus* spp. in MM. Additionally, the effect of *Klebsiella* spp. on buffalo milk should be noted in HM. On the other hand, *Pseudomonas* spp. is widely present in various milking methods; hence, it has significantly contributed to antibiotic resistance genes and virulence genes. Therefore, it is crucial to pay attention to their impact on the quality of raw milk during cold storage before processing. Our result will open the door for new research about this health concept.

## Figures and Tables

**Table 1:** Differences in basal nutrient composition, SCC and colony size of raw buffalo milk between two milking methods.

Milking Method	Protein (g/100 g)	Fat (g/100 g)	Total Solid (g/100 g)	Solids-Non-Fat g/100 g)	Lactose (g/100 g)	SCC (×10^4^/mL)	CFU (×10^4^ CFU/mL)
HM	4.55 ± 0.39 ^a^	7.33 ± 0.46 ^a^	17.81 ± 1.34 ^a^	9.77 ± 0.88 ^a^	4.99 ± 0.39 ^a^	45.85 ± 31.33 ^a^	37.06 ± 17.62 ^a^
MM	4.50 ± 0.29 ^a^	7.03 ± 0.68 ^a^	17.61 ± 0.76 ^a^	9.99 ± 0.44 ^a^	5.18 ± 0.20 ^a^	29.58 ± 15.56 ^a^	8.37 ± 8.37 ^b^

**Note:** All data shown were (mean ± standard deviation). The same lowercase letter in the shoulder scale represents a non-significant difference (*p* > 0.05), and different lowercase letters represent a significant difference (*p* < 0.05).

**Table 2:** Analysis of α diversity was conducted on raw buffalo milk samples from HM and MM groups.

Milking Method	Observed.	Simpson	Chao1	ACE	Shannon	Good’s Coverage
HM	830.4444 ± 49.9758 ^a^	0.8603 ± 0.0713 ^a^	852.363 ± 35.7754 ^a^	849.2941 ± 28.6194 ^a^	3.2567 ± 0.5710 ^a^	0.9998 ± 0.0003 ^a^
MM	665.1667 ± 71.3008 ^b^	0.7875 ± 0.0994 ^a^	777.734 ± 53.5937 ^b^	780.1879 ± 45.1084 ^b^	2.5035 ± 0.6112 ^b^	0.9982 ± 0.0011 ^b^

**Note:** All data shown were (mean ± standard deviation). The same lowercase letter in the shoulder scale represents a non-significant difference (*p* > 0.05), and different lowercase letters represent a significant difference (*p* < 0.05).

## Data Availability

All data generated or analyzed during this study are included in this published article (and its Supplementary Information files). The datasets generated and analyzed during the current study are available in the NCBI SRA repository submission number: PRJNA1084919. Data has not been released yet and will be available upon manuscript publication.

## Supplementary Materials

The following supporting information can be downloaded at: https://www.mdpi.com/article/10.3390/genes15081081/s1, Table S1: Evaluation statistics of sequencing data of 15 milk samples.
