# Supplementary material for: Meta-Genomic Analysis of Different Bacteria and Their Genomes Found in Raw Buffalo Milk Obtained in Various Farms Using Different Milking Methods
Source: Genes (Basel). 2024 Aug 15;15(8):1081. doi: 10.3390/genes15081081 (PMC11353964; doi:10.3390/genes15081081)
Supplement: Supplementary file 1 [file genes-15-01081-s001.zip › genes-3128251-supplementary.pdf]

Supplementary Table S1: Evaluation statistics of sequencing data of 15 milk samples.

Group	Sample ID	Raw data base(bp)	Clean data base(bp)	Number of Reads	GC(%)	Q20(%)	Q30(%)
MM	A1	7,841,790,600	98574324	660492	50.05%	93.02%	83.41%
MM	A2	7,109,118,600	542813348	3629456	48.30%	92.52%	80.15%
MM	A3	8,988,979,200	128440314	860238	49.38%	95.15%	87.40%
MM	A4	7,484,541,000	171674982	1148372	54.74%	96.33%	90.01%
MM	A5	8,563,971,600	173874032	1163550	50.82%	95.95%	89.13%
MM	A6	8,723,709,000	143371996	958724	50.91%	95.59%	88.11%
HM	B1	8,735,325,300	168758752	1129444	47.21%	96.14%	89.60%
HM	B2	10,050,748,500	703922664	4716314	54.92%	97.43%	92.28%
HM	B3	11,667,046,500	2092493338	14030032	55.64%	97.70%	92.94%
HM	B4	9,848,993,700	491072442	3291490	53.55%	97.36%	92.29%
HM	BN1	6,777,971,700	822588234	5495018	49.77%	94.34%	82.97%
HM	BN2	5,950,117,500	172629302	1158114	49.55%	90.82%	76.89%
HM	BN3	17,230,175,100	773192188	5182048	45.42%	94.97%	86.06%
HM	BN4	5,607,660,000	364757178	2439652	48.22%	93.29%	81.27%
HM	BN5	15,677,921,400	777190740	5192886	51.46%	95.96%	87.83%
Total		140,258,069,700	7,625,353,834	51,055,830			
AVER		9,350,537,980	508,356,922	3,403,722			

Note: Q20(%): Percentage of the base with a mass value greater than or equal to 20 in total base; Q30(%): Percentage of the base with a mass value greater than or equal to 30 in entire base; GC(%): GC content of a sample, namely the percentage of G and C type bases in total bases.
